# Erratum: Yoon, J.J., et al. *Dianthus superbus* Improves Glomerular Fibrosis and Renal Dysfunction in Diabetic Nephropathy Model. *Nutrients* 2019, *11*, 553

**DOI:** 10.3390/nu12061655

**Published:** 2020-06-03

**Authors:** Jung Joo Yoon, Ji Hun Park, Hye Jin Kim, Hong-Guang Jin, Hye Yoom Kim, You Mee Ahn, Youn Chul Kim, Ho Sub Lee, Yun Jung Lee, Dae Gill Kang

**Affiliations:** 1 Hanbang Cardio-Renal Syndrome Research Center, Wonkwang University, 460, Iksan-daero, Iksan, Jeonbuk 54538, Korea; mora16@naver.com (J.J.Y.); hyeyoomc@naver.com (H.Y.K.); aum2668@naver.com (Y.M.A.); host@wku.ac.kr (H.S.L.); 2 College of Oriental Medicine and Professional Graduate School of Oriental Medicine, Wonkwang University, 460, Iksan-daero, Iksan, Jeonbuk 54538, Korea; jihuncjstk@naver.com; 3 College of Pharmacy, Wonkwang University, Iksan 54538, Korea; mn1003@naver.com (H.J.K.); hg_jin1979@163.com (H.-G.J.); yckim@wku.ac.kr (Y.C.K.); 4 School of Pharmacy and Life Sciences, Jiujiang University, Jiujiang 332005, China

The authors have requested that the following changes be made to their paper [1]. 

Funding has been changed to follow as: “This work was supported by a National Research Foundation of Korea (NRF) grant funded by the Korean government (MSIP) (2017R1A5A2015805) (2017R1A2B4009884) (2015M3A9A5030620) (2018R1D1A1B07050720).” 

The authors apologize for any inconvenience caused to the readers by the change. The change does not affect the scientific results.
