# Supplementary material for: Dianthus superbus Improves Glomerular Fibrosis and Renal Dysfunction in Diabetic Nephropathy Model
Source: Nutrients. 2019 Mar 5;11(3):553. doi: 10.3390/nu11030553 (PMC6471502; doi:10.3390/nu11030553)
Supplement: Supplementary file 1 [file nutrients-11-00553-s001.pdf]

Supplementary Materials

1. Methods

1.1. Experimental Animals

Male db/db mice (C57BLKS/+Lepr^{db}) and age-matched nondiabetic db/m mice (C57BLKS/J) were purchased from Clea Japan (Tokyo, Japan). At 12 weeks of age, the mice were divided into five groups: db/m mice, db/db mice, db/m mice treated with aminoguanidine (AG, 20 mg/kg/day), db/db mice treated with DS-EA-low dose (DSL, 10 mg/kg/day), and db/db mice treated with DS-EA-high dose (DSH, 50 mg/kg/day). Body weight and water/food consumption were measured weekly, and blood glucose levels and glucose tolerance tests were measured every 4 weeks in all animals. At 12, 16, and 20 weeks of age, individual mice were placed in metabolic cages for 24 h of urine collection. The urine samples were stored at 4 °C until analysis. At the end of the experimental period, all the mice were sacrificed after 12 h of fasting, and blood samples were collected into 1-mg/ml ethylenediaminetetraacetic acid (EDTA)-coated tubes after being pulled out of the eyeball. The blood samples were centrifuged at 3000 rpm for 15 min at 4 °C, and plasma and erythrocyte were separated and then frozen at -70 °C until analysis. The thoracic aorta, kidney, liver, spleen, and pancreas were removed, washed, and then frozen at -70 °C until analysis. All procedures were approved by the Institutional Animal Care and Utilization Committee for Medical Science of Wonkwang University.

1.2. Monitoring of Renal Function

The mice of each group were maintained in separate metabolic cages for 2 days, allowing quantitative urine collections and measurements of water and food intake. Urine samples were collected for the determination of renal function parameter levels. Concentrations of ions were measured using an electrolyte analyzer (NOVA 5, Biochemical, Waltmam, MA).

1.3. Assessment of Renal Mesangial Cell Viability (MTT Assay)

To determine cell viability, 20 μl of MTT was added to a cell suspension for 4 h. After three washes with phosphate-buffered saline (PBS), the insoluble formazan product was dissolved in dimethyl sulfoxide. The optical density (OD) of each culture well was measured using a microplate reader (Multiskan; Thermo Labsystems, Franklin, MA) at 560 nm. The OD in control cells was considered to be 100% viable.

2. Results

2.1. Effect of DS-EA on Body Weight of db/db Mice

The body weight of the untreated db/db mice at 8–16 weeks of age was significantly higher than the control group mice. The db/db mice group showed a significant decrease in kidney weight compared to the control group. However, there was no significant difference in body weight between untreated db/db mice and DS-EA-treated db/db mice (Figure S1).

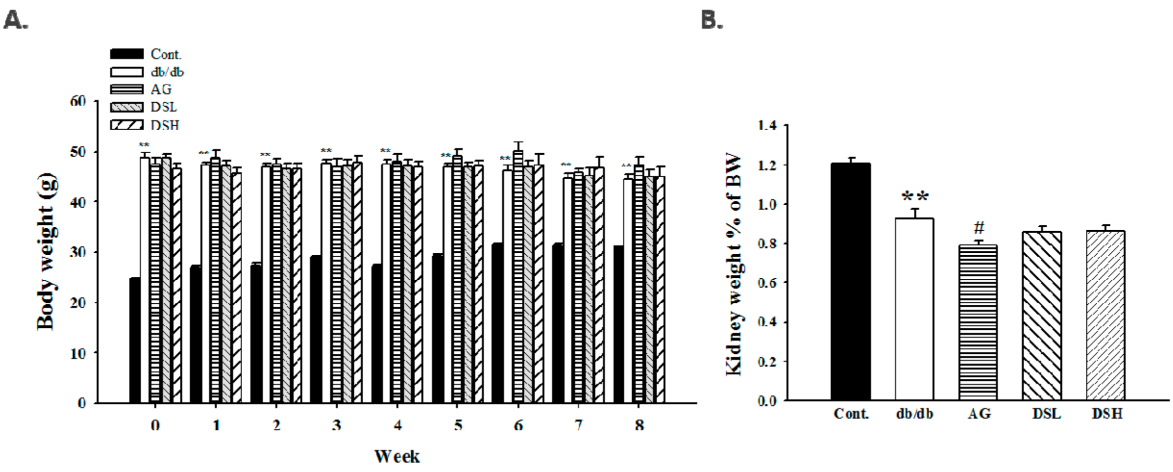

Figure S1. Effect of DS-EA on body weight (BW) **(A)** and kidney weight % of BW **(B)**. Values are expressed as mean ± S.E. ($n = 8$). ** $p < 0.01$ vs. Cont.; # $p < 0.05$, ## $p < 0.01$ vs. negative Cont.

2.2. Effect of DS-EA on Electrolytes

The urine sodium (Na), potassium (K), and chloride (Cl) volumes of the db/db mice group were significantly increased compared to the control group at 10–16 weeks. DS-EA-treated db/db mice had decreased electrolytes compared to the untreated db/db mice group during all the experimental period (Figure S2).

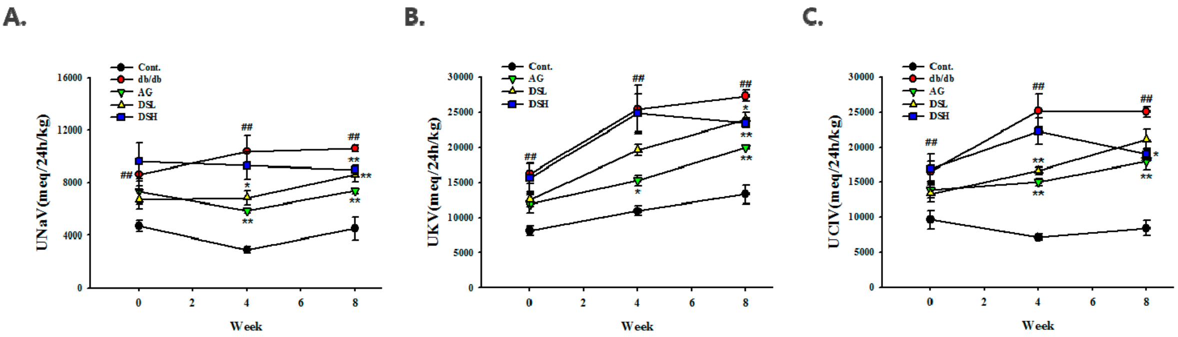

Figure S2. Effect of *Dianthus superbus* on UNaV **(A)**, UKV **(B)**, and UClV **(C)**. Values are expressed as mean ± S.E. ($n = 8$). ** $p < 0.01$ vs. Cont.; # $p < 0.05$, ## $p < 0.01$ vs. negative Cont.

2.3. Effect of DS-EA on Mesangial Cell Viability

The effect of DS-EA (0–50 μg/mL) on mesangial cell viability was evaluated by MTT assay. Cell viability assays revealed no obvious cytotoxic effects up to 20 μg/ml of DS-EA, but concentrations starting at 50 μg/ml caused a 64.5% reduction in cell viability.

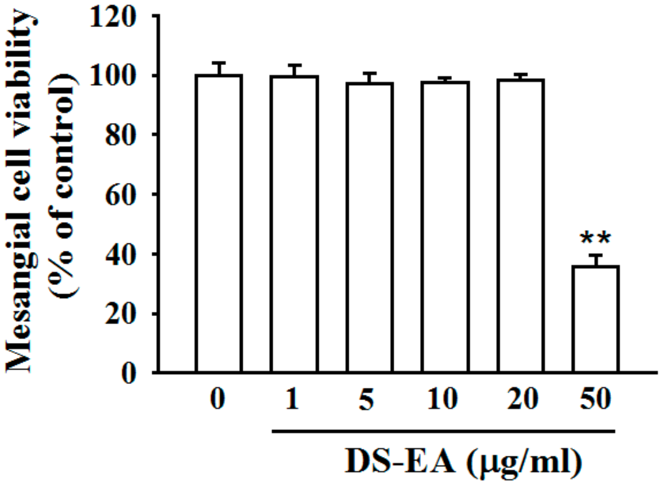

Figure S3. Human renal mesangial cell viability was assessed using an MTT assay. Results are expressed as the mean ± S.E. from five independent experiments. ** $p < 0.01$ vs. control.
